# Metabolomic Analysis Reveals Association between Decreased Ovarian Reserve and In Vitro Fertilization Outcomes

**DOI:** 10.3390/metabo14030143

**Published:** 2024-02-27

**Authors:** Na An, Min Zhang, Quan-Fei Zhu, Yao-Yu Chen, Yan-Ling Deng, Xiao-Ying Liu, Qiang Zeng, Yu-Qi Feng

**Affiliations:** 1 Department of Chemistry, Wuhan University, Wuhan 430072, China; 2017202030018@whu.edu.cn (N.A.); lacter@whu.edu.cn (Y.-Y.C.); 2 School of Bioengineering and Health, Wuhan Textile University, Wuhan 430200, China; yqfeng@whu.edu.cn; 3 Department of Occupational and Environmental Health, School of Public Health, Tongji Medical College, Huazhong University of Science and Technology, Wuhan 430030, China; d202181639@hust.edu.cn (M.Z.); yanling.deng@emory.edu (Y.-L.D.); m202175452@hust.edu.cn (X.-Y.L.); 4 Key Laboratory of Environment and Health, Ministry of Education & Ministry of Environmental Protection, State Key Laboratory of Environmental Health (Incubating), School of Public Health, Tongji Medical College, Huazhong University of Science and Technology, Wuhan 430030, China

**Keywords:** in vitro fertilization, diminished ovarian reserve, metabolomics, human serum

## Abstract

In vitro fertilization (IVF) is a highly effective treatment for infertility; however, it poses challenges for women with decreased ovarian reserve (DOR). Despite the importance of understanding the impact of DOR on IVF outcomes, limited research has explored this relationship, particularly using omics approaches. Hence, we conducted a study to investigate the association between DOR and IVF outcomes, employing a metabolomic approach. We analyzed serum samples from 207 women undergoing IVF treatment, including 89 with DOR and 118 with normal ovarian reserve (NOR). Our findings revealed that DOR was significantly associated with unfavorable IVF outcomes, characterized by a reduced oocyte count, lower embryo quality, and decreased rates of pregnancy and live births. Furthermore, we identified 82 metabolites that displayed significant alterations in DOR patients, impacting diverse metabolic pathways. Notably, a distinct panel of metabolites, including palmitic acid, stearic acid, LysoPC(9:0(CHO)/0:0), PC(18:0/9:0(CHO)), and PC(16:0/9:0(CHO)), exhibited discriminatory power between the DOR and NOR groups, showcasing a strong correlation with IVF outcomes. These findings emphasize the crucial role of metabolomic disruptions in influencing IVF outcomes among women with DOR.

## 1. Introduction

Owing to the deleterious impact of environmental pollution, psychological stress, and unhealthy lifestyles, infertility has witnessed a marked uptick in recent years and emerged as a pressing global health issue [1,2,3,4]. In a recent report published by the World Health Organization in 2022, it was estimated that the global prevalence of infertility stands at 17.5%, with the Western Pacific region exhibiting the highest rates, peaking at 23.2% [5,6]. Over the past two decades, China has witnessed a rise in infertility rates among those of reproductive age from 2.5–3.0% to 12.5–18.0% [7,8]. Consequently, infertility has posed a significant scientific challenge in the realm of life sciences in the present century.

Assisted reproductive technology (ART), specifically in vitro fertilization (IVF), is currently acknowledged as one of the most effective strategies for addressing infertility problems [9,10]. Despite significant progress in the field of IVF, several challenges persist in achieving optimal pregnancy rates. According to statistical data, the global success rate of live birth per IVF cycle is approximately 30% to 40% [10,11]. Factors such as age, body weight, ovarian reserve quantity and quality, unhealthy lifestyle choices, and exposure to environmental pollutants can all influence the likelihood of a successful outcome [10,12,13]. Among these factors, decreased ovarian reserve (DOR) presents a particularly formidable obstacle and frequently results in IVF failure [14,15,16]. Clinically, DOR is characterized by a decreased level of anti-Mullerian hormone (AMH), elevated levels of follicle-stimulating hormone (FSH), and/or a diminished antral follicle count (AFC) [17,18,19]. Patients with DOR are at risk of poor ovarian stimulation response, high rates of cycle cancellation, diminished oocyte yield, reduced pregnancy rates, and increased miscarriage rates following IVF treatment [20,21,22,23]. The estimated prevalence of DOR among cycles recorded in the Society for Assisted Reproductive Technology (SART) registry is projected to be 26% [14,24,25,26]. Approximately 6% of IVF cycles are terminated due to DOR, and this proportion has been progressively increasing in recent years [25]. The impact of DOR on IVF has received extensive attention; however, the etiology of reduced ovarian functional reserve is multifaceted, and the precise pathogenesis remains elusive, presenting challenges for the implementation of IVF technology and the selection of treatment modalities [27,28,29].

Metabolomics is widely regarded as the omics technology most closely associated with disease phenotypes within the omics family and has been recognized as a crucial component of precision medicine programs [30,31,32]. Employing a metabolomics approach for a systematic investigation of metabolic alterations in patients with DOR promises to identify specific metabolic pathways and key metabolites associated with DOR, elucidate the interactions between these metabolites and their correlation with IVF outcomes, and unveil the underlying pathophysiological mechanisms of DOR and potential intervention targets. Despite its potential, only a limited number of studies have conducted preliminary investigations into metabolic alterations in DOR [33,34,35,36,37,38,39], and omics studies on the correlation between DOR and IVF outcomes are still lacking.

**Figure 1:**
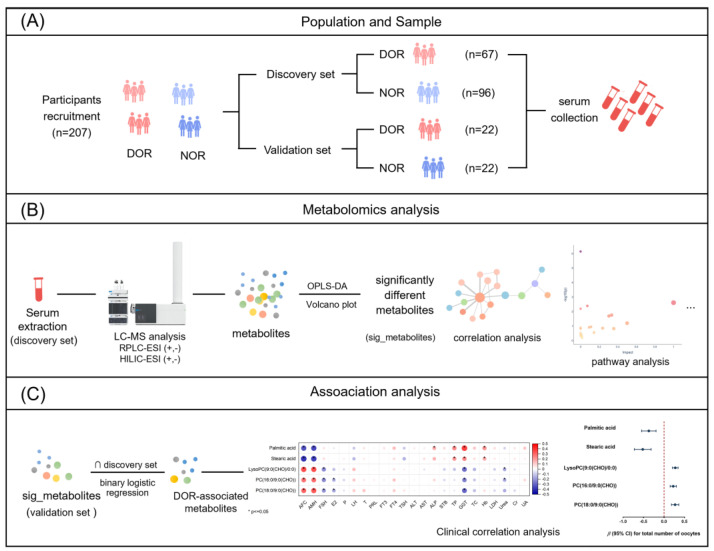
Overview of the study design. (**A**) Sample collection. (**B**) Metabolomic workflow. (**C**) Association workflow. DOR, diminished ovarian reserve; NOR, normal ovarian reserve.

In this investigation, we utilized an LC-MS-based (liquid chromatography-mass spectrometry) untargeted metabolomics approach to comprehensively profile the serum metabolites of 207 subjects undergoing IVF treatment, including 89 DOR cases and 118 controls (normal ovarian reserve, NOR), as depicted in Figure 1. We aimed to profile the metabolomic landscape between DOR and NOR groups in order to uncover metabolic pathways that are associated with DOR and identify a promising DOR-associated metabolite panel based on our high-resolution datasets. Furthermore, to better illustrate the role of the metabolites, we explored the association of DOR-associated metabolites with IVF outcomes.

## 2. Materials and Methods

### 2.1. Participants

Our study population was a subset of participants undergoing ART treatment enrolled in the Tongji Reproductive and Environmental (TREE) cohort. The ongoing TREE cohort was designed to explore the effects of environmental factors on couples’ reproductive health and early pregnancy outcomes, as previously described elsewhere [40,41,42]. Briefly, the couples visiting the Reproductive Medicine Center of Tongji hospital in Wuhan, China who planned to have a child were recruited from December 2018. Each subject was invited to finish a detailed questionnaire and provided biological samples after recruitment. By January 2020, a total of 2057 eligible women were recruited. All participants signed informed consent after understanding the study procedures, and the research protocol was approved by the Ethics Committee of Tongji Medical College.

In the TREE cohort, 622 women provided serum samples at enrollment. Among them, 275 women were excluded if they had at least one of the conditions that might affect ovarian reserve including chromosome abnormality, ovarian diseases (i.e., a history of ovarian surgery, ovarian cysts, polycystic ovarian morphology, and polycystic ovary syndrome (PCOS)), immune thrombocytopenic purpura, gynecological diseases (i.e., pelvic inflammatory disease, and endometriosis), and endocrine diseases (i.e., insulin resistance, hypogonadotropic hypogonadism, pituitary microadenoma, hyperprolactinemia, and thyroid disease). Additionally, 18 women who did not provide sufficient serum volumes for metabolomics analysis were excluded. The rest of the women were classified into two groups according to the following criteria: (1) DOR cases: women with a clinical diagnosis of DOR if their AFC ≤ 7, and/or AMH ≤ 1.1 ng/mL on day 2 to day 5 of the menstrual cycle; (2) controls: women with AFC > 7, AMH > 1.1 ng/mL, regular menstrual cycle and basal FSH < 10 IU/L on day 2 to day 5 of the menstrual cycle. Finally, a total of 163 subjects including 67 DOR patients and 96 controls were enrolled in the discovery set (Figure 1A). To derive the validation set, we randomly selected 22 pair participants in the TREE cohort who were not included in the discovery set [43]. The detailed participant selection flowchart is shown in Figure S1.

### 2.2. Chemicals and Reagents

Metabolite standards were purchased from Energy Chemical Co. (Shanghai, China), J&K (Beijing, China) and Sigma–Aldrich (St. Louis, MO, USA) reagent companies. Analytical-grade formic acid and ammonium formate were obtained from Sinopharm Chemical Reagent Co., Ltd. (Shanghai, China). LC-MS grade methanol (MeOH) and acetonitrile (ACN) were purchased from Merck (Darmstadt, Germany). The ultrapure water (H_2_O) used was prepared using a Milli-Q apparatus (Millipore, Bedford, MA, USA).

### 2.3. Serum Sample Collection and Preparation

Venous blood samples were collected from each woman before any medical intervention on day 2 to day 5 of the menstrual cycle. Following centrifugation at 3000 rpm for 10 min, serum samples were obtained. These serum samples were carefully dispensed into tubes and subsequently stored at −80 °C until further analysis. 

Each serum sample (100 μL) was subjected to extraction and deproteinization by adding 400 μL of cold MeOH. The mixture was vortexed for 30 s and stored at −20 °C for 20 min and then centrifuged at 13,000 rpm for 10 min. Afterward, the supernatant was collected and dried under nitrogen, following which it was dissolved in 150 μL MeOH/H_2_O (*v*/*v*, 5/5) prior to LC-MS analysis. Blank samples were also utilized for the experiment in which 100 μL H_2_O was employed instead of serum, and the other steps were the same as mentioned above.

To assess the precision of the overall analysis, a quality control (QC) sample was prepared through mixing an equal aliquot (20 μL) from each serum sample. In the course of the experiment, the serum samples were randomly analyzed, and the QC was analyzed every 10 samples to ensure and maintain consistent and accurate data quality.

### 2.4. LC-MS Analysis

Serum samples were analyzed using a UHPLC-Q-TOF MS system consisting of an Agilent 1290 Infinity II liquid chromatography system and an Agilent 6546 Q-TOF mass spectrometer (Agilent, Palo Alto, CA, USA) equipped with an Agilent Jet Stream electrospray ionization source (ESI, Turbo Ionspray). LC separation was performed using a Waters Acquity BEH C18 column (100 × 2.1 mm i.d., 1.7 μm) and a Waters Acquity BEH HILIC column (100 × 2.1 mm i.d., 1.7 μm). The C18 column was run at a flow rate of 0.4 mL/min, and the column temperature was 40 °C. A mobile phase comprising 0.1% formic acid aqueous solution (*v*/*v*, solvent A) and 0.1% formic acid ACN (*v*/*v*, solvent B) was used for both positive and negative ion modes. The gradient was as follows: 0–1 min, 2% B; 1–23 min, 2–98% B; 23–25 min, 98% B; 25–25.1 min, 98–2% B; 25.1–30 min, 2% B. 

Analysis of the HILIC column utilized a flow rate of 0.3 mL/min at 30 °C. The mobile phases consisting of 0.1% formic acid and 10 mM ammonium formate aqueous solution (*v*/*v*, solvent A) and 0.1% formic acid ACN (*v*/*v*, solvent B) were employed for both positive and negative ion modes. A gradient of 0–2 min, 95% B; 2–18 min, 95–60% B; 18–20 min, 60% B; 20–23 min, 60–95% B; 23–30 min, 95% B was applied. The injection volume was set at 5 μL.

The MS analysis was performed under a full scan mode of *m*/*z* 50–1000 with an acquisition rate of 2.5 spectra/s. The ESI parameters were set as follows: ion transfer tube temperature, 320 °C; fragmentor, 120 V; spray voltage, 3500 V for positive ion, 3000 V for negative ion; sheath gas, 11 L/min; sheath gas temperature, 350 °C; drying gas, 8 L/min; nebulizer, 35 psi. An auto MS/MS mode was used to acquire the MS2 spectra of significantly different metabolites. The MS scan rate was 3 spectra/s, and the MS/MS scan rate was 8 spectra/s. The MS2 fragment ions were acquired via collision-induced dissociation with collision energies of 10, 20, 30, and 40, an intensity threshold of 10,000, a maximum of 6 precursor per cycle, and a 0.2 min dynamic exclusion time.

### 2.5. Data Processing

Raw data were acquired using the Agilent 6546 MassHunter Workstation software (version 10.1, Agilent Technologies). The raw data (.d format) were initially converted to abf files by ABF_Converter. Following this, MS-DIAL software (version 4.70) was utilized for peak detection, deconvolution, peak alignment, blank subtraction, and LOESS normalization to generate a comprehensive feature list. Afterwards, the web-based MS-FLO tool was employed to remove isotope peaks, adduct ions, duplicate peaks, and contaminant ions [44]. To remove metabolites with high missing values, we applied the 80% rule, and the missing values were filled with 1/5 of the minimum value for the remaining metabolites [45]. For metabolites detected by two or more platforms, the values with the lowest relative standard deviation (RSD) in QC samples were kept, and metabolites with RSDs less than 30% in QC samples were used for further analysis. Prior to statistical analysis, the data were log-transformed to approximate normal distribution. 

Feature-based Global Natural Products Social Molecular Networking (GNPS, https://gnps.ucsd.edu/ProteoSAFe/static/gnps-splash.jsp (accessed on 9 August 2016)) and SIRIUS 4.9.15 (https://bio.informatik.uni-jena.de/sirius/ (accessed on 18 March 2019)) were used for metabolite annotation via MS2 spectra acquired by auto-MS/MS mode. Significantly different metabolites were annotated through standard confirmation, public MS2 database matching, and MS/MS interpretation, with the annotation level complying with the Metabolomics Standards Initiative grade [46,47].

### 2.6. IVF Outcome Assessment

The IVF outcomes were assessed from the couple’s first ART treatment cycle, as previously described in detail [48]. At the treatment cycle, women underwent specific IVF treatment protocols based on age, infertility diagnosis, and ovarian response: (a) long luteal-phase gonadotropin-releasing hormone (GnRH) agonist; (b) GnRH antagonist; and (c) others such as minimal stimulation IVF protocol. When more than two follicles matured, women underwent human chorionic gonadotropin (hCG) injection. Oocyte retrieval was performed by the specialized physician 34–36 h after the trigger shot. The retrieved oocytes were counted and assessed for maturity under the microscope by an embryologist. The mature (MII) oocytes were used for insemination. Fertilization was determined to be normal when two polar nuclei and two pronuclei (2PN) appeared in the fertilized oocyte 16–18 h after insemination. The 2PN cleavage zygotes are those normal fertilized oocytes that can continue to divide after the fertilization. Embryos were classified as high-quality if they had 4–5 cells on day 2, 7–10 cells on day 3, and fragmentation less than 10%. Fertilization rate was calculated by dividing the number of normal fertilized oocytes by the number of MII oocytes. The 2PN cleavage rate was defined as the number of 2PN cleavage zygotes divided by the number of normal fertilized oocytes. High-quality embryo rate was the ratio of the number of high-quality embryos to the number of 2PN cleavage zygotes. Implantation success was defined as a serum β-hCG concentration of more than 10 IU/L on day 14 after embryo transfer. Clinical pregnancy was defined as the presence of an intrauterine pregnancy confirmed by ultrasound 3–4 weeks after embryo transfer. Live birth was defined as the delivery of a live neonate on or after 28 weeks of gestation.

### 2.7. Statistical Analysis

The descriptive statistics were performed for the study population demographic and clinical characteristics. Differences in these characteristics between the NOR controls and DOR cases were examined by Chi-square tests for categorical variables and Wilcoxon’s rank sum tests for continuous variables. Multivariate statistical analysis including principal component analysis (PCA) and orthogonal partial least squares discriminant analysis (OPLS-DA) were performed using SIMCA14.1 (Umea, Sweden). Variable important in the projection (VIP) values of the OPLS-DA model were used to recognize significant variables that contributed notably to classification. A 200-permutation test was conducted to evaluate the reliability and accuracy of the OPLS-DA model. 

Univariate analysis, metabolic pathway analysis, enrichment analysis, and correlation analysis were performed through MetaboAnalyst 5.0, and volcano plot, box-plot, and chord diagrams were visualized by Origin. Correlation network analysis was plotted using Cytoscape3.9.1. Binary logistic regression analysis was executed using IBM SPSS 25.0 software to establish the biomarker model, and the receiver-operating characteristic curve (ROC) was used to evaluate the results of the regression analysis. The ROC curve was plotted using the random forest model of PyCharm Community Edition 201.1.3. 

We used generalized linear regression (GLM) models to evaluate the associations between DOR and IVF outcomes of their first cycle and between DOR-associated metabolites and IVF outcomes of their first cycle. A Poisson distribution and log link function were applied to count outcomes (e.g., the total number of oocytes, MII oocytes, 2PN oocytes, 2PN cleavage zygotes, and high-quality embryos), a binomial distribution and logit link function were applied for proportional outcomes (i.e., fertilization rate, 2PN cleavage rate, and high-quality embryo rate), and a binary distribution with a logit link function was applied for the binary clinical outcomes (i.e., implantation success, clinical pregnancy, and live birth). According to our prior knowledge [48], the following covariates were included in the final model: age (continuous), body mass index (BMI, <25.0 kg/m^2^ vs. ≥25.0 kg/m^2^), passive smoking status (yes vs. no), alcohol status (never vs. ever/current), educational level (less than high school vs. high school and above), income (≤5000 vs. >5000 yuan/month), and infertility diagnosis (female factor, male factor, mixed factor vs. unexplained). The GLM models were fitted using R (version 4.2.1), and a two-sided *p*-value less than 0.05 was considered statistically significant.

## 3. Results

### 3.1. Participant Characteristics

Table 1 presents the characteristics of the IVF participants in each set. In this study, a total of 207 women undergoing IVF treatment were enrolled, following stringent inclusion and exclusion criteria outlined in the experimental section (Materials and Methods). Among these participants, there were 89 cases of DOR and 118 controls, with mean ages of 33.6 and 32.0 years, respectively. Moreover, the gonadotropin-releasing hormone (GnRH) antagonist was the primary treatment protocol for DOR cases, whereas the long GnRH antagonist was mainly used for the controls. In both the discovery set and the validation set, women with DOR had significantly lower total AFC and AMH levels and poorer IVF outcomes (e.g., total number of oocytes) than the controls. The other characteristics were comparable between the DOR cases and controls in both sets.

### 3.2. Metabolomic Profiling

**Figure 2:**
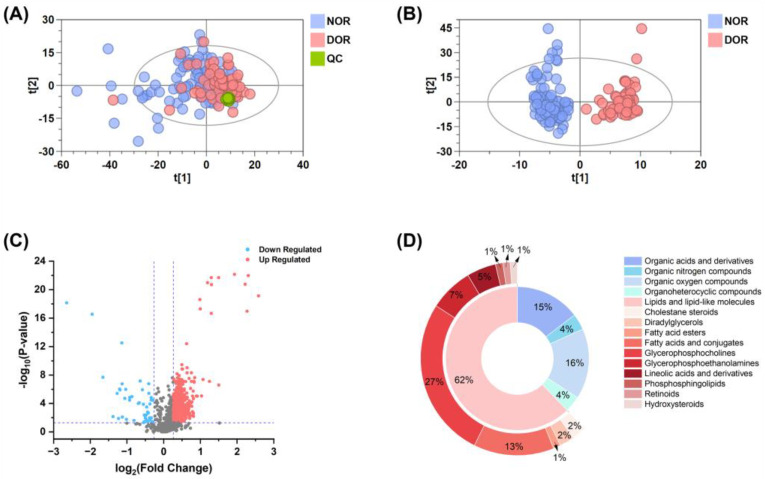
Identification of differential metabolomics profiles in serum between DOR and NOR. Score plots of PCA (**A**) and OPLS-DA (**B**) based on the combinational data of RPLC-ESI(+) TOF-MS, RPLC-ESI(−) TOF-MS, HILIC-ESI(+) TOF-MS, and HILIC-ESI(−) TOF-MS from the discovery set. The pink circles represent DOR; the blue circles represent NOR; the green circles represent QC samples. (**C**) Volcano plot, down-regulated, up-regulated, and not significantly changed metabolites in DOR compared to NOR are marked in blue, red, and grey, respectively. (**D**) Distribution of metabolites across super/sub-classes in the discovery set.

To obtain a comprehensive metabolomic landscape between DOR and NOR groups, large-scale untargeted metabolomics profiling was performed on serum samples from the discovery set using reversed-phase liquid chromatography (RPLC) and hydrophilic interaction chromatography (HILIC) combined with Q-TOF MS, in both positive and negative ionization modes (Figure 1B). Following data processing, 1494, 860, 987, and 458 features were detected in RPLC-ESI(+) TOF-MS, RPLC-ESI(−) TOF-MS, HILIC-ESI(+) TOF-MS, and HILIC-ESI(−) TOF-MS, respectively. In order to evaluate the precision of our overall analysis, we employed PCA analysis on these detected features. The resulting PCA score plots showed that the QC samples were tightly clustered, confirming that the acquired metabolomics data were reliable and accurate (Figure 2A).

### 3.3. Screening and Annotation of Significantly DOR-Associated Metabolites

To discern the metabolic distinctions between DOR and NOR groups, we conducted multivariate and univariate statistical analyses. The OPLS-DA score plots clearly demonstrated a distinct separation between DOR patients (pink circles) and NOR controls (blue circles, as shown in Figure 2B). Additionally, the robustness of the OPLS-DA model was confirmed by a 200-permutation test, indicating that it was not overfitting and had strong predictability (as illustrated in Figure S2). The volcano plot (Figure 2C) showed a significant dysregulation of metabolites in DOR patients compared to NOR controls, with a considerable number of metabolites exhibiting up- or down-regulation (FDR *p* < 0.05, FC > 1.2). Overall, a total of 344 metabolites exhibited significant alterations based on the multiple criteria of FDR *p* < 0.05, FC > 1.2, and VIP > 1. In DOR patients, 314 metabolites were found to be up-regulated and 30 metabolites to be down-regulated, indicating significant metabolic alterations in this IVF population.

Subsequently, these metabolites with significant differences were annotated to gain a better understanding of their potential functions. As a result, 82 metabolites were confidently annotated (Table S1), including 28 confirmed by standards (level 1), 40 identified through matching MS2 spectra to public databases (level 2), and 14 classified based on MS2 spectra (level 3). The differential metabolites were classified into five superclasses based on the chemical classification system of ClassyFire (Figure 2D), including lipids and lipid-like molecules (62%), organic oxygen compounds (16%), organic acids and derivatives (15%), organoheterocyclic compounds (4%), and organic nitrogen compounds (4%). Remarkably, lipids and lipid-like molecules constituted the largest portion of the differential metabolites. These diverse lipid molecules can be subcategorized into 10 subclasses, including glycerophosphocholines (27%), fatty acids and conjugates (13%), glycerophosphoethanolamines (7%), and lineolic acids and derivatives (5%). These results highlight the structural diversity of differential metabolites involved in DOR metabolic abnormalities. A representative heatmap of the above different significant metabolites is shown in Figure S3.

### 3.4. Correlation Analysis of Significantly Different Metabolites

**Figure 3:**
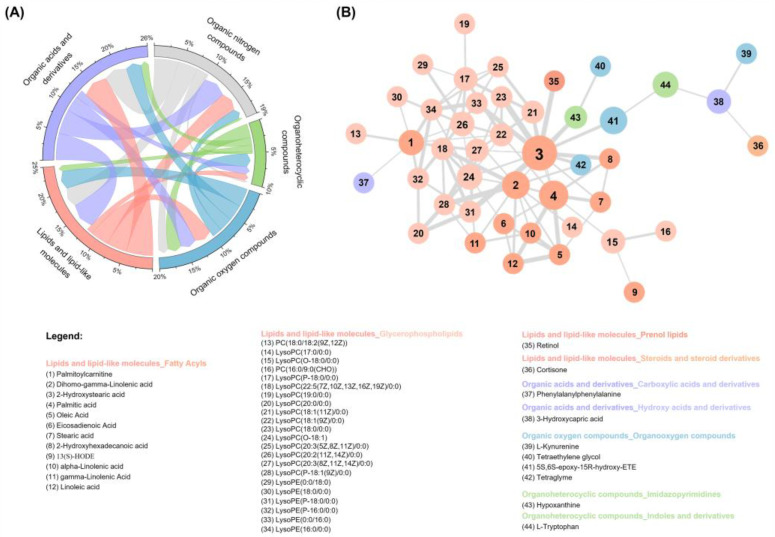
Associations between differential metabolites. (**A**) Chord diagram displaying the Pearson correlation of the superclasses for the differential metabolites between DOR and NOR. (**B**) Debiased sparse partial correlation network analysis illustrating the differential correlation between individual significantly different metabolites. The node size of each metabolite is reflected by its betweenness centrality (how frequently a metabolite occurs on the shortest paths between other metabolites). The thickness of the lines connecting metabolites is scaled in relation to the -lg (adjust *p*-values). Metabolite names are listed in the legend.

A chord diagram and correlation network analyses were employed to explore the interrelationships among differential metabolites (level 1–2) based on their normalized intensity, aiming to gain a more comprehensive understanding of their interconnectedness. The chord diagram, as depicted in Figure 3A, revealed that organic acids and derivatives, lipids and lipid-like molecules, and organic nitrogen compounds had a correlation (r > 0.4), while the correlation between organic oxygen compounds and organoheterocyclic compounds and other groups was comparatively weaker. The correlation network analysis showed that the lipids and lipid-like molecules were the most prominent entities in the molecular network (Figure 3B). Specifically, 2-hydroxystearic acid, a fatty acyls metabolite, was identified as the central node in the correlation network. It bridged the altered metabolites and exhibited a strong correlation with other metabolites. Glycerophospholipids accounted for the largest proportion in the molecular network and displayed dense interactions (Figure 3B). These findings suggest that DOR is closely associated with abnormal lipid and organic acid metabolism.

### 3.5. Pathway Analysis and Enrichment Analysis for DOR-Associated Metabolites

**Figure 4:**
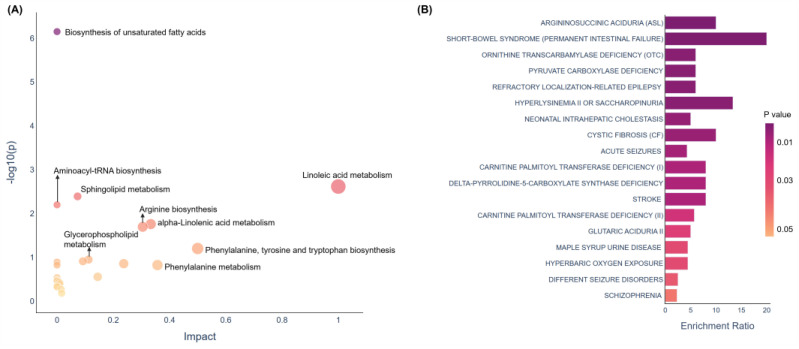
(**A**) Pathway analysis of significantly different metabolites in DOR according to the KEGG pathway. (**B**) Human disease states that correlated with DOR-related metabolites on the basis of published metabolomics data.

Pathway analysis and enrichment analysis were performed using MetaboAnalyst 5.0, based on the differential metabolites identified in the discovery set, with reference to the Kyoto Encyclopedia of Genes and Genomes (KEGG) database. Our KEGG pathway analysis revealed that the metabolic dysregulation induced by DOR was primarily associated with unsaturated fatty acid biosynthesis, linoleic acid metabolism, sphingolipid metabolism, aminoacyl-tRNA biosynthesis, alpha-linolenic acid metabolism, arginine biosynthesis, phenylalanine, tyrosine and tryptophan biosynthesis, glycerophospholipid metabolism, and phenylalanine metabolism (Figure 4A). Notably, the dysregulation of metabolic pathways related to unsaturated fatty acids was remarkably significant, indicating a potential target for therapeutic intervention. Furthermore, the enrichment analysis indicated that numerous metabolites associated with DOR were implicated in the pathogenesis of diverse human disorders, such as argininosuccinic aciduria (ASL), short-bowel syndrome (permanent intestinal failure), and ornithine transcarbamylase deficiency (Figure 4B).

### 3.6. Development of a DOR-Associated Metabolite Panel

To evaluate the dependability of significant differential metabolites discovered in the discovery set, untargeted metabolomic analysis was also conducted on serum samples from 22 DOR and 22 NOR IVF participants of the validation set (Figure S4A–D). By conducting a comparative analysis of differential metabolites between the validation and discovery sets, we identified 10 DOR-associated metabolites that exhibit consistent variation trends across both sets. These metabolites include palmitic acid, stearic acid, glucose, hypoxanthine, cholesterol sulfate, indoxyl sulfate, LysoPC(9:0(CHO)/0:0), PC(18:0/9:0(CHO)), PC(16:0/9:0(CHO)), and phenylalanylphenylalanine. 

**Figure 5:**
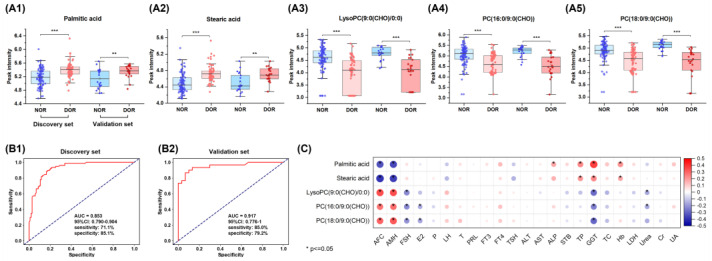
Performance of the biomarker signature for the diagnosis of DOR. (**A**) Boxplots of the five DOR-associated metabolites in the discovery set and validation set: (**A1**) palmitic acid, (**A2**) stearic acid, (**A3**) LysoPC(9:0(CHO)/0:0), (**A4**) PC(16:0/9:0(CHO)), and (**A5**) PC(18:0/9:0(CHO)). **, 0.001 < *p* < 0.01; ***, *p* < 0.001. (**B**) Receiver operating characteristics curves and corresponding area under the curve (AUC), confidence interval, and the sensitivity and specificity of the biomarker signature for differentiating DOR from NOR. (**B1**) Discovery set; (**B2**) Validation set. (**C**) Heatmap of the Spearman correlation coefficients between five DOR-associated metabolites and clinical parameters. The colors in the heatmap represent the positive (represented by red) or negative correlation (represented by blue). *, *p* ≤ 0.05. E2, Estradiol; P, Progesterone; LH, Luteinizing hormone; T, Testosterone; PRL, Prolactin; FT3, Free triiodothyronine; FT4, Free thyroxine; TSH, Thyroid-stimulating hormone; ALT, Alanine aminotransferase; AST, Aspertate aminotransferase; ALP, Alkaline phosphatase; STB, Serum total bilirubin; TP, Total protein; GGT, Glutamyl transpeptidase; TC, Total cholesterol; Hb, Hemoglobin; LDH, Lactate dehydrogenase; Cr, Creatinine; UA, Uric acid.

Subsequently, binary logistic regression analysis was performed on the 10 significant differential metabolites, resulting in the identification of five metabolites with higher DOR prediction accuracy: palmitic acid, stearic acid, LysoPC(9:0(CHO)/0:0), PC(18:0/9:0(CHO)), and PC(16:0/9:0(CHO)). In the serum of DOR patients, levels of palmitic acid and stearic acid were significantly elevated, while LysoPC (9:0 (CHO)/0:0), PC (18:0/9:0(CHO)), and PC (16:0/9:0(CHO)) levels were significantly reduced (Figure 5A). These five metabolites were defined as a DOR-associated metabolite panel.

We utilized the PLS-DA machine learning model to construct ROC curves for evaluating the diagnostic performance of the above metabolite panel. Our ROC analysis demonstrated that the areas under the curve (AUC) for this panel in both discovery and validation sets were 0.853 and 0.917, respectively, with a specificity of 85.1% and 79.2%, as well as a sensitivity of 71.1% and 85.0% (Figure 5B), indicating a favorable diagnostic ability for IVF women with DOR.

To enhance our comprehension of the relationship between serum metabolite profile and the DOR phenotype, we investigated the correlation between these five metabolites and various clinical parameters, encompassing AFC, AMH, FSH, estradiol (E2), progesterone (P), and luteinizing hormone (LH). The Spearman rank analysis revealed a negative correlation between fatty acid stearic acid and palmitic acid with ovarian function-related indicators AFC and AMH, while showing a positive correlation with TP, GGT, and Hb. Meanwhile, the three phospholipid metabolites demonstrated a positive correlation with AFC and AMH while exhibiting a negative correlation with FSH, E2, and GGT. Additionally, these metabolite levels did not show significant correlations with clinical parameters, such as P and LH, in the subjects (Figure 5C).

### 3.7. Associations with IVF Outcomes

We first investigated the association between DOR and IVF outcomes. The findings, as shown in Table 2, indicate significant negative associations between DOR status and various IVF outcomes, such as the total number of oocytes, MII oocytes, 2PN oocytes, 2PN cleavage zygotes, high-quality embryos, implantation, clinical pregnancy, and live birth (all *p* < 0.05). Compared to the control group, DOR cases exhibited decreases of 0.77 (95% CI: −0.88, −0.67) in the total number of oocytes, 0.76 (95% CI: −0.88, −0.65) in MII oocytes, 0.84 (95% CI: −0.98, −0.70) in 2PN oocytes, 0.85 (95% CI: −0.99, −0.71) in 2PN cleavage zygotes, and 0.99 (95% CI: −1.19, −0.78) in high-quality embryos. Furthermore, the probability of successful conception, clinical pregnancy, and live birth were reduced by 77% (RR = 0.23, 95% CI: 0.11, 0.49), 78% (RR = 0.22, 95% CI: 0.10, 0.46), and 71% (RR = 0.29, 95% CI: 0.14, 0.60) among DOR cases compared to the control group, respectively. However, there were no significant associations between DOR status and fertilization rate, 2PN cleavage rate, and high-quality embryo rate. 

**Figure 6:**
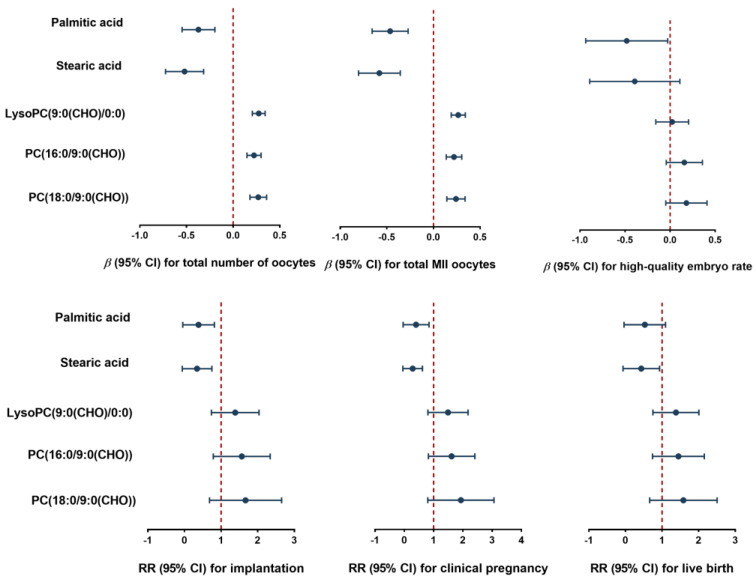
The associations between serum metabolites and IVF outcomes based on GLM models. The models were adjusted by age (continuous), body mass index (BMI, <25.0 kg/m^2^ vs. ≥25.0 kg/m^2^), passive smoking status (yes vs. no), alcohol status (never vs. ever/current), educational level (less than high school vs. high school and above), income (≤5000 vs. >5000 yuan/month) and infertility diagnosis (female factor, male factor, mixed factor vs. unexplained). Data for count and proportional outcomes are presented as adjusted β (95% CI) and for binary outcomes as adjusted RR (95% CI).

We further explored the relationship between this DOR-associated metabolite panel and IVF outcomes. As depicted in Figure 6 and Table S2, serum palmitic acid and stearic acid were negatively associated with IVF outcomes, whereas LysoPC(9:0(CHO)/0:0), PC(18:0/9:0(CHO)), and PC(16:0/9:0(CHO)) were positively associated with IVF outcomes. After adjusting for covariates, one-unit increase in serum palmitic acid was significantly associated with a 0.37 (95% CI: −0.55, −0.20), 0.46 (95% CI: −0.66, −0.27), 0.50 (95% CI: −0.73, −0.27), 0.48 (95% CI: −0.71, −0.25), 0.71 (95% CI: −1.03, −0.39), and 0.48 (95% CI: −0.94, −0.03) decrease in total number of oocytes, MII oocytes, 2PN oocytes, 2PN cleavage zygotes, high-quality embryos, and high-quality embryo rates, respectively; one-unit increase in serum stearic acid was significantly associated with a 0.52 (95% CI: −0.73, −0.32), 0.58 (95% CI: −0.80, −0.36), 0.65 (95% CI: −0.91, −0.38), 0.61 (95% CI: −0.88, −0.34), and 0.81 (95% CI: −1.19, −0.44) decrease in total number of oocytes, MII oocytes, 2PN oocytes, 2PN cleavage zygotes, and high-quality embryos. Moreover, 78% (RR = 0.22, 95% CI: 0.06, 0.87) and 76% (RR = 0.24, 95% CI: 0.06, 0.90) lower probabilities of successful implantation clinical pregnancy were estimated for one-unit increase in serum palmitic acid, respectively; lower successful implantation (RR = 0.19, 95% CI: 0.03, 0.80), clinical pregnancy (RR = 0.15, 95% CI: 0.03, 0.66), and live birth (RR = 0.24, 95% CI: 0.06, 0.99) were estimated for one-unit increase in serum stearic acid. Additionally, higher levels of serum LysoPC(9:0(CHO)/0:0), PC(18:0/9:0(CHO)), and PC(16:0/9:0(CHO)) were positively associated with the total number of oocytes, MII oocytes, 2PN oocytes, 2PN cleavage zygotes, and high-quality embryos, but no significant associations were found with high-quality embryo rates, implantation, clinical pregnancy, and live birth. There were null associations between these metabolites and fertilization rate and 2PN cleavage rate.

## 4. Discussion

Our study aimed to investigate the effects of DOR on IVF outcomes from a metabolomic perspective. We conducted a comprehensive metabolomic evaluation of 207 women undergoing IVF treatment, including 89 DOR cases and 118 NOR controls, using large-scale untargeted metabolomics analysis. Metabolic characteristics revealed significant disparities between DOR cases and NOR controls, as evidenced by 82 metabolites significantly altered in serum samples, such as lipids and lipid molecules, organic acids and derivatives. Through binary logistic regression analysis, we identified a DOR-associated metabolite panel (stearic acid, palmitic acid, PC(18:0/9:0(CHO)), PC(16:0/9:0(CHO)), and LysoPC(9:0(CHO)/0:0)) that exhibited good discrimination between DOR and NOR groups. Moreover, it is crucial to highlight that these specific metabolites demonstrated a robust association with IVF outcomes.

The presence of DOR has been shown to be linked with ovarian hypo-response, low pregnancy rates, and a high miscarriage rate in IVF treatment [18,19,35]. Our study, involving 207 women undergoing IVF treatment, reinforced these observations. The DOR group displayed a significant decrease in the number and quality of oocytes compared to the NOR group. Specifically, there was a reduction of 0.76 to 0.99 in the total number of oocytes, MII oocytes, 2PN oocytes, 2PN cleavage zygotes, and high-quality embryos (Table 2). The decline in oocyte quantity and quality among DOR patients led to the failure of clinical pregnancy and live birth. The data contribute to the ever-increasing evidence that DOR negatively affects fertility and pregnancy in IVF. 

Stearic acid and palmitic acid are the predominant saturated fatty acids present in serum [49]. Previous studies have shown that elevated levels of these acids have a negative impact on human oocyte and ovarian follicle function, ultimately resulting in infertility [49,50]. Additionally, heightened levels of stearic and palmitic acids in follicular fluid are linked to impaired oocytes, diminished fertilization rates, and compromised embryo quality [51,52]. Notably, these acids’ presence in cow follicular fluid have been identified as potential biomarkers for bovine infertility [53]. Consistent with these findings, we noted similar trends in DOR patients undergoing IVF, with serum samples exhibiting elevated levels of palmitic and stearic acids (Figure 5A1,A2), and a high reversed correlation between them and the quantity and quality of oocyte (total number of oocytes and MII oocytes), as well as the growth and development of the embryo (implantation success, clinical pregnancy, and live birth, Figure 6). Our results highlighted that stearic acid and palmitic acid may be responsible for poor IVF outcomes in patients with DOR.

Our findings indicate a substantial decrease in the serum levels of three phospholipid aldehydes, namely PC(16:0/9:0(CHO)), PC(18:0/9:0(CHO)), and LysoPC(9:0(CHO)/0:0), in DOR women undergoing IVF in comparison to NOR controls (Figure 6 and Table S2). Importantly, these phospholipid aldehyde levels displayed a significant positive correlation with oocyte maturation. It is recognized that aldehydes possess antioxidant properties [54], and therefore, we propose that the decline in phospholipid aldehydes observed in DOR women may be attributed to lipid peroxidation. Moreover, we posit that these three phospholipid aldehydes have the potential to be utilized as pharmacological agents to augment oocyte development; however, it is imperative to substantiate their effectiveness through rigorous preclinical animal experiments followed by subsequent clinical trials.

We observed that a wide range of metabolic disturbances occurred in DOR patients, including sphingolipid metabolism, arginine biosynthesis, glycerophospholipid metabolism, aminoacyl-tRNA biosynthesis, alpha-linolenic acid metabolism, phenylalanine, tyrosine and tryptophan biosynthesis, and the biosynthesis of unsaturated fatty acids and linoleic acid metabolism.

Among these, the alterations in the metabolic pathways of polyunsaturated fatty acids (PUFAs) are particularly significant, such as the biosynthesis of unsaturated fatty acids, alpha-linolenic acid metabolism, and linoleic acid metabolism. These findings suggest a close association between PUFAs and the development of DOR. Previous studies have shown that PUFA oxidation is an important energy source for oocyte maturation and division [52,55,56]. However, excessive PUFA oxidation can increase the level of reactive oxygen species (ROS), leading to dysfunction in the mitochondria and endoplasmic reticulum, ultimately impairing oocyte development [57,58,59,60]. The up-regulation of the three PUFA-related metabolic pathways suggests that DOR patients may experience more severe oxidative stress damage. 

Disorders in sphingolipid metabolism, particularly involving the sphingosine-1-phosphate (S1P) signaling pathway, have been reported to be closely associated with the development and progression of gynecological conditions [61,62,63]. Previous studies have shown that infertile women with PCOS and severe endometriosis exhibit significantly heightened levels of S1P in comparison to healthy women [61,62,63]. Similar alterations were observed in the serum of women with DOR (Figure S5). S1P is a crucial lipid signaling molecule that plays a pivotal role in preventing oocyte apoptosis and promoting oocyte maturation [64,65,66]. Therefore, it is hypothesized that the S1P signaling pathway may be highly activated in these infertile patients, but further investigation is required to elucidate the underlying mechanisms.

Glycerophospholipids serve as the primary constituents of mammalian cell membranes, exerting a pivotal role in signal transduction, cellular proliferation, protein function, and other essential biological processes [57,67,68]. In line with these previous studies, we also observed an up-regulation of the glycerophospholipid metabolic pathway in women with DOR. Glycerophospholipid metabolites accounted for the largest proportion of significantly altered differential metabolites, as evidenced by our correlation network analysis (Figure 3B). Overall, our study indicates that the development of DOR may be driven by abnormal lipid metabolism, with disorders in glycerophospholipid, sphingolipid, and fatty acid metabolic pathways. These observations indirectly emphasize the significance of lipid metabolism as one of the most extensively researched areas in reproductive studies.

We also noted disruptions in three biosynthetic pathways related to amino acids, including aminoacyl-tRNA biosynthesis, arginine biosynthesis, and the phenylalanine, tyrosine, and tryptophan biosynthesis pathways. DOR patients exhibit elevated levels of various amino acids in their serum, such as L-phenylalanine, L-lysine, L-arginine, and L-tryptophan (Figure S6). Similar observations have been reported in previous studies on women with PCOS and DOR [60,69,70]. Disruptions in amino acid metabolism may affect the balance of cellular osmotic pressure, ultimately leading to impaired oocyte function [71,72].

This study presents a comprehensive evaluation of serum metabolites, offering valuable insights into the metabolomic differences between women with DOR and NOR undergoing IVF. However, there are certain limitations that should be acknowledged. Firstly, the observed metabolic alterations could be attributed to DOR or other factors. Further mechanistic studies are necessary to determine whether the identified metabolic changes are the causal factors or consequences of DOR. Secondly, larger sample sizes are often desirable to enhance the reliability of the findings and to account for potential confounding factors. Thirdly, it is important to note that the study participants were recruited from a single medical center in China, which may limit the generalizability of the findings to other populations and regions. In addition, considering the several DOR-associated metabolites we identified belong to the lipid class, conducting additional lipidomics analysis on the serum samples would provide further insights into DOR. Exploring this avenue in future research holds significant potential.

## 5. Conclusions

In conclusion, we utilized a large-scale untargeted metabolomics approach to uncover the comprehensive metabolic profiles of women with DOR. Our findings revealed significant disruptions in multiple metabolic pathways, including lipid and amino acid metabolism pathways, among women with DOR undergoing IVF. Furthermore, we identified five metabolites consisting of two fatty acids and three phospholipid aldehydes that can effectively distinguish DOR from NOR populations, while also exhibiting a robust correlation with their IVF outcomes. Collectively, these findings furnish novel metabolomic evidence supporting the detrimental impact of DOR on fertility and pregnancy outcomes in IVF treatment. The identification of specific DOR-associated metabolites provide crucial insights and data support for investigating the underlying mechanisms, which may facilitate the development of targeted interventions to optimize IVF outcomes for patients with DOR.

## Figures and Tables

**Table 1:** Demographic and clinical characteristics of the participants in each set [Mean ± SD or n (%)].

Characteristics	Discovery Set		Validation Set		*p*-Value ^a^	*p*-Value ^b^
	Non-DOR (N = 96)	DOR (N = 67)	Non-DOR (N = 22)	DOR (N = 22)		
Age (years old)	31.9 ± 2.7	34.0 ± 6.9	32.5 ± 3.0	32.5 ± 3.0	0.233	0.999
Age at menarche (years old)	13.4 ± 1.1	13.4 ± 1.3	13.2 ± 0.8	12.9 ± 1.2	0.555	0.603
Infertility duration (years)	3.8 ± 2.6 (3 missing)	3.3 ± 3.2 (1 missing)	4.4 ± 3.6 (1 missing)	4.3 ± 2.5	0.041	0.816
BMI (kg/m^2^)	21.8 ± 3.2	22.9 ± 3.8	22.4 ± 2.6	22.4 ± 2.8	0.078	0.916
<25.0	80 (83.3%)	48 (71.6%)	18 (81.8%)	18 (81.8%)	0.074	0.999
≥25.0	16 (16.7%)	19 (28.4%)	4 (18.2%)	4 (18.2%)		
Race						
Han	93 (96.9%)	65 (97.0%)	21 (95.5%)	22 (100.0%)	0.999	0.999
Others	3 (3.1%)	2 (3.0%)	1 (4.5%)	0 (0%)		
Marital status						
First marriage	83 (86.5%)	54 (80.6%)	18 (81.8%)	20 (90.9%)	0.315	0.664
Remarriage	13 (13.5%)	13 (19.4%)	4 (18.2%)	2 (9.1%)		
Gravidity						
Yes	55 (57.3%)	20 (29.9%)	8 (36.4%)	11 (50.0%)	0.001	0.361
No	41 (42.7%)	47 (70.1%)	14 (63.6%)	11 (50.0%)		
Parity						
Nulliparous	78 (81.3%)	46 (68.7%)	12 (54.5%)	19 (86.4%)	0.064	0.021
Parous	18 (18.8%)	21 (31.3%)	10 (45.5%)	3 (13.6%)		
Household income (yuan/month)						
≤5000	50 (52.1%)	38 (56.7%)	12 (54.5%)	11 (50.0%)	0.559	0.763
>5000	46 (47.9%)	29 (43.3%)	10 (45.5%)	11 (50.0%)		
Passive smoking status						
Yes	18 (18.8%)	21 (31.3%)	7 (31.8%)	7 (31.8%)	0.064	0.999
No	78 (81.3%)	46 (68.7%)	15 (68.2%)	15 (68.2%)		
Alcohol status						
Never	78 (81.3%)	62 (92.5%)	21 (95.5%)	21 (95.5%)	0.042	0.999
Ever/Current	18 (18.8%)	5 (7.5%)	1 (4.5%)	1 (4.5%)		
Educational level						
Less than high school	35 (36.5%)	27 (40.3%)	11 (50.0%)	9 (40.9%)	0.619	0.545
High school and above	61 (63.5%)	40 (59.7%)	11 (50.0%)	13 (59.1%)		
Exercise frequency						
Never	31 (32.3%)	27 (40.3%)	11 (50.0%)	12 (52.3%)	0.575	0.029
Occasionally	49 (51.0%)	30 (44.8%)	11 (50.0%)	6 (27.3%)		
Frequently	16 (16.7%)	10 (14.9%)	0 (0.0%)	4 (18.2%)		
Total AFC (n)	13.6 ± 4.7	4.6 ± 2.2	12.5 ± 3.4	4.8 ± 1.7	<0.001	<0.001
FSH (IU/L)	6.9 ± 1.4 (1 missing)	9.1 ± 3.1 (1 missing)	7.8 ± 1.4	9.9 ± 5.6	<0.001	0.222
E2 (pg/mL)	42.9 ± 17.2 (1 missing)	49.1 ± 28.2 (2 missing)	35.4 ± 12.1	45.6 ± 31.2 (2 missing)	0.195	0.208
AMH (ng/mL)	4.0 ± 2.2 (1 missing)	1.1 ± 0.6 (1 missing)	3.7 ± 2.3	1.1 ± 0.7	<0.001	<0.001
Infertility diagnosis of couples						
Female factor	35 (36.4%)	50 (74.6%)	9 (40.9%)	18 (81.8%)	<0.001	0.011
Male factor	24 (25.0%)	0 (0.0%)	5 (22.7%)	0 (0.0%)		
Mixed factor	21 (21.8%)	17 (25.4%)	5 (22.7%)	4 (18.2%)		
Unexplained factor	16 (16.6%)	0 (0.0%)	3 (13.6%)	0 (0.0%)		
Treatment protocol						
Long GnRH agonist	55 (71.4%)	3 (4.6%)	15 (75.0%)	2 (9.5%)	<0.001	<0.001
GnRH antagonist	20 (25.9%)	40 (61.5%)	5 (25.0%)	16 (76.1%)		
Others	2 (2.4%)	22 (33.7%)	0 (0.0%)	3 (14.2%)		
IVF outcomes						
Total number of oocytes retrieved	13.8 ± 6.4	5.9 ± 3.8	12.9 ± 6.5	6.2 ± 3.8	<0.001	0.001
Mature (MII) oocytes retrieved	11.3 ± 5.6	5.1 ± 3.2	10.9 ± 5.7	5.1 ± 3.6	<0.001	0.001
Normal fertilized (2PN) oocytes	8.3 ± 4.7	3.6 ± 2.9	8.1 ± 4.8	3.0 ± 2.5	<0.001	<0.001
2PN cleavage zygotes	8.1 ± 4.6	3.5 ± 3.0	8.0 ± 4.7	3.0 ± 2.4	<0.001	<0.001
High-quality embryos	4.3 ± 3.2	1.7 ± 1.7	4.5 ± 2.9	1.5 ± 1.4	<0.001	<0.001
Fertilization rate (%) ^c^	72.2 ± 18.5	66.9 ± 31.4	73.7 ± 17.2	63.7 ± 28.0	0.807	0.237
2PN cleavage rate (%) ^d^	97.3 ± 7.4	95.3 ± 19.2	98.6 ± 5.1	98.6 ± 6.4	0.173	0.594
High-quality embryo rate (%) ^e^	52.4 ± 24.3	50.0 ± 33.9	63.2 ± 25.4	45.9 ± 36.0	0.635	0.086
Implantation success ^f^	57 (77.0%)	25 (49.0%)	16 (84.2%)	8 (44.4%)	0.001	0.011
Clinical pregnancy ^g^	53 (71.6%)	19 (37.3%)	15 (78.9%)	7 (38.9%)	<0.001	0.013
Live birth ^h^	46 (62.2%)	17 (33.3%)	13 (68.4%)	6 (33.3%)	0.002	0.033

Abbreviations: BMI, body mass index; AFC: antral follicle count; FSH: follicle-stimulating hormone; E2: estradiol; AMH: anti-müllerian hormone. There were five women missing infertility duration, two missing FSH, five missing E2, and two missing AMH. There were 183 women who entered IVF cycles and 21 women who entered IVF cycles without transferring embryos. *p*-value was calculated by Chi-square tests (categorical variables) or Wilcoxon’s rank sum tests (continuous variables). ^a^ The comparison of DOR and non-DOR groups in the discovery set. ^b^ The comparison of DOR and non-DOR groups in the validation set. ^c^ Value was calculated as the number of 2PN oocytes divided by MII oocytes. ^d^ Value was calculated as the number of 2PN cleavage zygotes divided by 2PN oocytes. ^e^ Value was calculated as the number of high-quality embryos divided by 2PNcleavage zygotes. ^f^ Implantation success was defined as a positive pregnancy test (serum HCG level > 10 IU/L) 14 days after embryo transfer. ^g^ Clinical pregnancy was defined as the presence of a gestational sac and fetal heartbeat in the uterus confirmed by ultrasound 3–4 weeks after embryo transfer. ^h^ Live birth was defined as the delivery of a live neonate on or after 28 weeks of gestation.

**Table 2:** Associations between DOR status and IVF outcomes based on GLM models ^a^.

Characteristic	NOR	DOR	*p*-Value
**β (95% CI)**			
Total number of oocytes	ref	−0.77 (−0.88, −0.67)	<0.001
MII oocytes	ref	−0.76 (−0.88, −0.65)	<0.001
2PN oocytes	ref	−0.84 (−0.98, −0.70)	<0.001
2PN cleavage zygotes	ref	−0.85 (−0.99, −0.71)	<0.001
High-quality embryos	ref	−0.99 (−1.19, −0.78)	<0.001
Fertilization rate	ref	−0.25 (−0.51, 0.01)	0.06
2PN cleavage rate	ref	−0.22 (−1.17, 0.74)	0.66
High-quality embryo rate	ref	−0.28 (−0.58, 0.01)	0.06
**RR (95% CI)**			
Implantation success	ref	0.23 (0.11, 0.49)	<0.001
Clinical pregnancy	ref	0.22 (0.10, 0.46)	<0.001
Live birth	ref	0.29 (0.14, 0.60)	<0.001

^a^ All models were adjusted by age (continuous), body mass index (BMI, <25.0 kg/m^2^ vs. ≥25.0 kg/m^2^), passive smoking status (yes vs. no), alcohol status (never vs. ever/current), educational level (less than high school vs. high school and above), income (≤5000 vs. >5000 yuan/month) and infertility diagnosis (female factor, male factor, mixed factor vs. unexplained).

## Data Availability

All data that support the findings of the study are within the manuscript or in Supplemental Materials.

## Supplementary Materials

The following supporting information can be downloaded at: https://www.mdpi.com/article/10.3390/metabo14030143/s1, Table S1: Annotated significantly different metabolites in DOR serum; Table S2: Associations between serum metabolites and IVF outcomes based on GLM models; Figure S1: Participants’ selection flowchart; Figure S2: Cross-validation plot with a permutation test repeated 200 times of the OPLS-DA score plot; Figure S3: Hierarchical clustering of each sample data set showing the differentially expressed metabolites; Figure S4: Validation of potential metabolic biomarkers for the diagnosis of DOR; Figure S5: Boxplots of the relative abundances of sphingosine-1-phosphate in the DOR and NOR groups within the discovery set; Figure S6: Boxplots of the relative abundances of L-arginine, L-lysine, L-phenylalanine, and L-tryptophan in the DOR and NOR groups within the discovery set.
