# Supplementary material for: Metabolomic Analysis Reveals Association between Decreased Ovarian Reserve and In Vitro Fertilization Outcomes
Source: Metabolites. 2024 Feb 27;14(3):143. doi: 10.3390/metabo14030143 (PMC10971940; doi:10.3390/metabo14030143)

Supporting information

Metabolomic Analysis Reveals Association between Decreased Ovarian Reserve and In vitro Fertilization Outcomes

Na An [1,2,†], Min Zhang [3,4,†], Quan-Fei Zhu [2,*], Yao-Yu Chen[1], Yan-Ling Deng [3,4], Xiao-Ying Liu [3,4], Qiang Zeng [3,4,*], Yu-Qi Feng [2]

[1] Department of Chemistry, Wuhan University, Wuhan 430072, China;
[2] School of Bioengineering and Health, Wuhan Textile University, Wuhan 430200, China; yqfeng@whu.edu.cn
[3] Department of Occupational and Environmental Health, School of Public Health, Tongji Medical College, Huazhong University of Science and Technology, Wuhan 430030, China
[4] Key Laboratory of Environment and Health, Ministry of Education & Ministry of Environmental Protection, State Key Laboratory of Environmental Health (Incubating), School of Public Health, Tongji Medical College, Huazhong University of Science and Technology, Wuhan 430030, China

* Correspondence: qf_zhu@whu.edu.cn (Q.-F.Z.); zengqiang506@hust.edu.cn (Q.Z.)
† These authors contributed equally to this work.

The supporting information includes following items:

Page S3-5	Table S1 Annotated significantly different metabolites in DOR serum
Page S6	Table S2 Associations between serum metabolites and IVF outcomes based on GLM models
Page S7	Figure S1 Participants' selection flowchart.
Page S8	Figure S2 Cross-validation plot with a permutation test repeated 200 times of the OPLS-DA score plot.
Page S9	Figure S3 Hierarchical clustering of each sample data set showing the differentially expressed metabolites.
Page S10	Figure S4 Validation of potential metabolic biomarkers for the diagnosis of DOR.
Page S11	Figure S5 Boxplots of the relative abundances of sphingosine-1-phosphate in the DOR and NOR groups within the discovery set.
Page S12	Figure S6 Boxplots of the relative abundances of typical amino acids in the DOR and NOR groups within the discovery set.

Table S1 Annotated significantly different metabolites in DOR serum.

Name	*m/z*	RT	Mode	VIP	FC	P-value	Formula	HMDB ID	Subclass	Superclass	Level
2-Hydroxystearic acid	299.2588	20.75	RPLC-ESI(-)	2.1	1.41	3.62E-10	C18H36O3	HMDB0010737	Fatty acids and conjugates	Lipids and lipid-like molecules	1
Stearic acid	283.264	22.44	RPLC-ESI(-)	2.0	1.69	4.91E-09	C18H36O2	HMDB0000827	Fatty acids and conjugates	Lipids and lipid-like molecules	1
2-Hydroxypalmitic acid	271.2278	18.81	RPLC-ESI(-)	1.9	1.34	1.19E-08	C16H32O3	HMDB0031057	Fatty acids and conjugates	Lipids and lipid-like molecules	1
Palmitic acid	255.2332	20.88	RPLC-ESI(-)	1.8	1.66	7.66E-07	C16H32O2	HMDB0000220	Fatty acids and conjugates	Lipids and lipid-like molecules	1
Oleic Acid	281.2491	21.11	RPLC-ESI(-)	1.4	1.48	1.63E-04	C18H34O2	HMDB0000207	Fatty acids and conjugates	Lipids and lipid-like molecules	1
Dihomo-gamma-Linolenic Acid	307.263	20.39	RPLC-ESI(+)	1.2	1.27	9.92E-04	C20H34O2	HMDB0002925	Fatty acids and conjugates	Lipids and lipid-like molecules	1
Eicosadienoic Acid	307.2639	21.40	RPLC-ESI(-)	1.1	1.51	1.13E-04	C20H36O2	HMDB0005060	Fatty acids and conjugates	Lipids and lipid-like molecules	1
16-Hydroxypalmitic acid	271.2277	14.87	RPLC-ESI(-)	1.2	1.36	1.05E-04	C16H32O3	HMDB0006294	Fatty acids and conjugates	Lipids and lipid-like molecules	1
Linoleic acid	279.2336	19.83	RPLC-ESI(-)	1.5	1.36	1.07E-04	C18H32O2	HMDB0000673	Lineolic acids and derivatives	Lipids and lipid-like molecules	1
gamma-Linolenic Acid	279.2318	18.87	RPLC-ESI(+)	1.0	1.38	2.48E-04	C18H30O2	HMDB0003073	Lineolic acids and derivatives	Lipids and lipid-like molecules	1
alpha-Linolenic acid	279.2323	18.70	RPLC-ESI(+)	1.4	1.29	3.28E-04	C18H30O2	HMDB0001388	Lineolic acids and derivatives	Lipids and lipid-like molecules	1
13(S)-HODE	295.2276	15.45	RPLC-ESI(-)	1.3	1.65	6.25E-06	C18H32O3	HMDB0004667	Lineolic acids and derivatives	Lipids and lipid-like molecules	1
PC(16:0/9:0(CHO))	650.4398	8.42	HILIC-ESI(+)	1.7	0.46	1.24E-06	C33H64NO9P		Glycerophosphocholines	Lipids and lipid-like molecules	1
Sphingosine-1-phosphate	378.2411	14.14	RPLC-ESI(-)	1.3	1.29	4.06E-06	C18H38NO5P	HMDB0000277	Phosphosphingolipids	Lipids and lipid-like molecules	1
Cortisone	405.1915	8.53	RPLC-ESI(-)	1.2	1.30	1.32E-05	C21H28O5	HMDB0002802	Hydroxysteroids	Lipids and lipid-like molecules	1
L-Lysine	145.0985	12.14	HILIC-ESI(-)	1.3	1.55	3.48E-06	C6H14N2O2	HMDB0000182	Amino acids, peptides, and analogues	Organic acids and derivatives	1
Phytosphingosine	3.18E+02	11.87	RPLC-ESI(+)	1.8	0.42	3.89E-06	C18H39NO3	HMDB0004610	Amino acids, peptides, and analogues	Organic acids and derivatives	1
L-Arginine	349.2313	11.73	HILIC-ESI(+)	1.0	1.28	7.52E-06	C6H14N4O2	HMDB0000517	Amino acids, peptides, and analogues	Organic acids and derivatives	1
Phenylalanylphenylalanine	313.1567	6.08	RPLC-ESI(+)	1.1	1.41	2.35E-05	C18H20N2O3	HMDB0013302	Amino acids, peptides, and analogues	Organic acids and derivatives	1
L-Phenylalanine	166.0865	2.37	RPLC-ESI(+)	1.2	1.60	2.21E-04	C9H11NO2	HMDB0000159	Amino acids, peptides, and analogues	Organic acids and derivatives	1
Glycyl-Phenylalanine	223.108	3.54	RPLC-ESI(+)	1.1	1.38	2.33E-04	C11H14N2O3	HMDB0028848	Amino acids, peptides, and analogues	Organic acids and derivatives	1
Citrulline	176.103	10.15	HILIC-ESI(+)	1.1	1.21	1.19E-04	C6H13N3O3	HMDB0000904	Amino acids, peptides, and analogues	Organic acids and derivatives	1
3-Hydroxycapric acid	187.1339	11.13	RPLC-ESI(-)	1.1	1.24	1.51E-02	C10H20O3	HMDB0002203	Medium-chain hydroxy acids and derivatives	Organic acids and derivatives	1
Sphingosine	300.2898	14.21	RPLC-ESI(+)	1.5	1.42	1.66E-06	C18H37NO2	HMDB0000252	Amines	Organic nitrogen compounds	1
Spermidine	146.1652	0.55	RPLC-ESI(+)	1.5	1.86	9.16E-06	C7H19N3	HMDB0001257	Amines	Organic nitrogen compounds	1

D-Glucose	203.0532	2.64	HILIC-ESI(+)	1.1	0.55	1.10E-05	C6H12O6	HMDB0000122	Carbohydrates and carbohydrate conjugates	Organic oxygen compounds	1
Hypoxanthine	159.0281	2.68	HILIC-ESI(+)	1.1	0.79	1.10E-02	C5H4N4O	HMDB0000157	Purines and purine derivatives	Organoheterocyclic compounds	1
L-Tryptophan	205.0974	7.18	HILIC-ESI(+)	1.3	1.20	1.70E-03	C11H12N2O2	HMDB0000929	Indolyl carboxylic acids and derivatives	Organoheterocyclic compounds	1
Palmitoylcarnitine	400.3428	15.94	RPLC-ESI(+)	1.0	1.34	4.56E-04	C23H46NO4	HMDB0000222	Fatty acid esters	Lipids and lipid-like molecules	2
DG(18:1(9Z)/18:1(9Z)/0:0)	643.5277	5.34	HILIC-ESI(+)	1.1	1.28	5.60E-04	C39H72O5	HMDB0007218	Diradylglycerols	Lipids and lipid-like molecules	2
AEG(o-16:2/18:2)	575.5045	5.37	HILIC-ESI(+)	1.0	1.22	7.32E-03	C37H66O4		Diradylglycerols	Lipids and lipid-like molecules	2
LysoPC(9:0(CHO)/0:0)	412.2101	10.42	HILIC-ESI(+)	2.1	0.36	2.16E-08	C17H34NO8P		Glycerophosphocholines	Lipids and lipid-like molecules	2
LysoPC(19:0/0:0)	538.3870	19.07	RPLC-ESI(+)	1.9	1.38	5.52E-08	C27H56NO7P		Glycerophosphocholines	Lipids and lipid-like molecules	2
LysoPC(18:0/0:0)	568.3622	9.21	HILIC-ESI(-)	1.8	1.28	1.13E-07	C26H54NO7P	HMDB0010384	Glycerophosphocholines	Lipids and lipid-like molecules	2
PC(18:0/9:0(CHO))	678.4719	8.33	HILIC-ESI(+)	1.8	0.46	1.79E-07	C35H68NO9P		Glycerophosphocholines	Lipids and lipid-like molecules	2
LysoPC(20:0/0:0)	552.4028	20.05	RPLC-ESI(+)	1.6	1.37	5.29E-07	C28H58NO7P	HMDB0010390	Glycerophosphocholines	Lipids and lipid-like molecules	2
LysoPC(O-18:1)	508.3768	17.02	RPLC-ESI(+)	1.6	1.37	8.52E-07	C26H54NO6P		Glycerophosphocholines	Lipids and lipid-like molecules	2
LysoPC(20:2(11Z,14Z)/0:0)	548.371	16.74	RPLC-ESI(+)	1.4	1.39	9.90E-07	C28H54NO7P	HMDB0010392	Glycerophosphocholines	Lipids and lipid-like molecules	2
LysoPC(17:0/0:0)	510.3565	17.10	RPLC-ESI(+)	1.6	1.47	1.66E-06	C25H52NO7P	HMDB0012108	Glycerophosphocholines	Lipids and lipid-like molecules	2
LysoPC(P-18:1(9Z)/0:0)	506.3608	17.07	RPLC-ESI(+)	1.4	1.32	2.48E-06	C26H52NO6P	HMDB0010408	Glycerophosphocholines	Lipids and lipid-like molecules	2
LysoPC(O-18:0/0:0)	510.3926	18.60	RPLC-ESI(+)	1.5	1.30	1.14E-05	C26H56NO6P	HMDB0011149	Glycerophosphocholines	Lipids and lipid-like molecules	2
LysoPC(P-18:0/0:0)	508.3769	18.60	RPLC-ESI(+)	1.4	1.28	1.29E-05	C26H54NO6P	HMDB0013122	Glycerophosphocholines	Lipids and lipid-like molecules	2
LysoPC(22:5(7Z,10Z,13Z,16Z,19Z)/0:0)	570.3565	15.80	RPLC-ESI(+)	1.3	1.42	5.21E-05	C30H52NO7P	HMDB0010403	Glycerophosphocholines	Lipids and lipid-like molecules	2
PC(18:0/18:2(9Z,12Z))	786.5998	8.85	HILIC-ESI(+)	1.3	1.30	6.05E-05	C44H84NO8P	HMDB0008039	Glycerophosphocholines	Lipids and lipid-like molecules	2
LysoPC(20:3(5Z,8Z,11Z)/0:0)	546.3563	16.07	RPLC-ESI(+)	1.3	1.34	3.62E-04	C28H52NO7P	HMDB0010393	Glycerophosphocholines	Lipids and lipid-like molecules	2
LysoPC(18:1(11Z)/0:0)	566.3464	16.25	RPLC-ESI(-)	1.1	1.28	1.09E-03	C26H52NO7P	HMDB0010385	Glycerophosphocholines	Lipids and lipid-like molecules	2
LysoPC(20:3(8Z,11Z,14Z)/0:0)	546.355	15.77	RPLC-ESI(+)	1.0	1.25	1.27E-03	C28H52NO7P	HMDB0010394	Glycerophosphocholines	Lipids and lipid-like molecules	2
LysoPC(18:1(9Z)/0:0)	566.3469	16.59	RPLC-ESI(-)	1.1	1.27	2.58E-04	C26H52NO7P	HMDB0002815	Glycerophosphocholines	Lipids and lipid-like molecules	2
LysoPE(0:0/18:0)	480.3095	17.62	RPLC-ESI(-)	1.7	1.41	1.06E-07	C23H48NO7P	HMDB0011129	Glycerophosphoethanolamines	Lipids and lipid-like molecules	2
LysoPE(18:0/0:0)	480.3098	18.01	RPLC-ESI(-)	1.7	1.36	3.53E-07	C23H48NO7P	HMDB0011130	Glycerophosphoethanolamines	Lipids and lipid-like molecules	2
LysoPE(P-16:0/0:0)	436.2834	16.58	RPLC-ESI(-)	1.4	1.28	7.13E-06	C21H44NO6P	HMDB0011152	Glycerophosphoethanolamines	Lipids and lipid-like molecules	2
LysoPE(P-18:0/0:0)	464.3148	18.54	RPLC-ESI(-)	1.4	1.30	2.66E-05	C23H48NO6P	HMDB0240598	Glycerophosphoethanolamines	Lipids and lipid-like molecules	2
LysoPE(16:0/0:0)	452.2784	16.06	RPLC-ESI(-)	1.2	1.31	3.77E-04	C21H44NO7P	HMDB0011503	Glycerophosphoethanolamines	Lipids and lipid-like molecules	2
LysoPE(0:0/16:0)	452.2779	15.68	RPLC-ESI(-)	1.0	1.31	5.23E-04	C21H44NO7P	HMDB0011473	Glycerophosphoethanolamines	Lipids and lipid-like molecules	2

Retinol	269.2268	19.45	RPLC-ESI(+)	1.7	1.28	1.33E-06	C20H30O	HMDB0000305	Retinoids	Lipids and lipid-like molecules	2
Cholesterol sulfate	465.3043	0.97	HILIC-ESI(-)	1.6	1.52	8.48E-08	C27H46O4S	HMDB0000653	Cholestane steroids	Lipids and lipid-like molecules	2
5alpha-Cholestanone	369.352	0.70	HILIC-ESI(+)	1.2	1.34	1.67E-03	C27H46O	HMDB0000871	Cholestane steroids	Lipids and lipid-like molecules	2
N,N-Dimethylarginine	203.1509	12.15	HILIC-ESI(+)	1.9	1.25	7.99E-08	C8H18N4O2	HMDB0001539	Amino acids, peptides, and analogues	Organic acids and derivatives	2
Glycylvaline	173.0932	8.43	HILIC-ESI(-)	1.2	1.63	1.26E-04	C7H14N2O3	HMDB0028854	Amino acids, peptides, and analogues	Organic acids and derivatives	2
Histidyltryptophan	342.1565	2.92	RPLC-ESI(+)	1.1	1.39	3.80E-04	C17H19N5O3	HMDB0028896	Amino acids, peptides, and analogues	Organic acids and derivatives	2
Indoxyl sulfate	212.0023	0.99	HILIC-ESI(-)	1.2	1.39	2.56E-03	C8H7NO4S	HMDB0000682	Arylsulfates	Organic acids and derivatives	2
Lauryldiethanolamine	274.2749	5.12	HILIC-ESI(+)	1.5	0.79	1.46E-05	C16H35NO2		Amines	Organic nitrogen compounds	2
L-Kynurenine	209.0924	2.28	RPLC-ESI(+)	1.3	1.24	1.86E-05	C10H12N2O3	HMDB0000684	Carbonyl compounds	Organic oxygen compounds	2
5S,6S-epoxy-15R-hydroxy-ETE	209.1385	3.80	RPLC-ESI(+)	3.6	3.81	7.09E-23	C9H20O5	HMDB0062236	Ethers	Organic oxygen compounds	2
Tetraglyme	223.1542	4.69	RPLC-ESI(+)	2.9	2.45	2.05E-22	C10H22O5	HMDB0258894	Ethers	Organic oxygen compounds	2
Tetraethylene glycol	195.1228	3.00	RPLC-ESI(+)	2.8	2.47	2.01E-21	C8H18O5	HMDB0094708	Ethers	Organic oxygen compounds	2
Pentaethylene glycol	2.39E+02	3.67	RPLC-ESI(+)	2.8	2.45	2.22E-17	C10H22O6	HMDB0256256	Ethers	Organic oxygen compounds	2
Acisoga	185.1287	3.27	RPLC-ESI(+)	1.3	1.28	1.01E-03	C9H16N2O2	HMDB0061384	N-alkylpyrrolidines	Organoheterocyclic compounds	2
-	445.3323	20.89	RPLC-ESI(-)	1.3	1.35	7.90E-04	C28H46O4		Fatty acids and conjugates	Lipids and lipid-like molecules	3
-	417.3008	18.93	RPLC-ESI(-)	1.1	1.30	4.76E-03	C26H42O4		Fatty acids and conjugates	Lipids and lipid-like molecules	3
-	391.2848	18.61	RPLC-ESI(-)	1.1	1.21	1.28E-02	C24H40O4		Fatty acids and conjugates	Lipids and lipid-like molecules	3
-	548.3720	17.07	RPLC-ESI(+)	1.7	1.44	1.97E-07	C28H54NO7P		Glycerophosphocholines	Lipids and lipid-like molecules	3
-	506.3607	15.77	RPLC-ESI(+)	1.6	1.32	4.40E-06	C26H52NO6P		Glycerophosphocholines	Lipids and lipid-like molecules	3
-	554.3461	17.10	RPLC-ESI(-)	1.5	1.40	5.70E-06	C26H54NO9P		Glycerophosphocholines	Lipids and lipid-like molecules	3
-	536.4073	18.87	RPLC-ESI(+)	1.2	1.37	1.26E-04	C28H58NO6P		Glycerophosphocholines	Lipids and lipid-like molecules	3
-	253.1646	4.37	RPLC-ESI(+)	3.5	4.95	1.07E-22	C11H24O6		Ethers	Organic oxygen compounds	3
-	267.1804	5.07	RPLC-ESI(+)	3.2	2.83	2.05E-22	C12H26O6		Ethers	Organic oxygen compounds	3
-	223.1541	4.58	RPLC-ESI(+)	2.9	2.29	1.04E-21	C10H22O5		Ethers	Organic oxygen compounds	3
-	281.1951	5.82	RPLC-ESI(+)	2.2	1.99	5.10E-18	C13H28O6		Ethers	Organic oxygen compounds	3
-	372.2595	5.83	RPLC-ESI(+)	1.7	1.42	1.42E-09	C16H34O8		Ethers	Organic oxygen compounds	3
-	386.2751	6.49	RPLC-ESI(+)	1.4	1.31	9.12E-07	C17H36O8		Ethers	Organic oxygen compounds	3
-	546.3853	8.49	RPLC-ESI(+)	1.1	1.26	2.12E-05	C25H52O11		Ethers	Organic oxygen compounds	3

m/z, mass-to-charge; RT, retention time; VIP, variable important in the projection; FC, fold change.

Table S2 Associations between serum metabolites and IVF outcomes based on GLM models[a].

Characteristic	Palmitic acid	Stearic acid	LysoPC(9:0(CHO)/0:0)	PC(16:0/9:0(CHO))	PC(18:0/9:0(CHO))
**β (95% CI)**					
Total number of oocytes	-0.37 (-0.55, -0.20)	-0.52 (-0.73, -0.32)	0.27 (0.21, 0.34)	0.22 (0.15, 0.30)	0.27 (0.18, 0.36)
MII oocytes	-0.46 (-0.66, -0.27)	-0.58 (-0.80, -0.36)	0.27 (0.19, 0.34)	0.22 (0.14, 0.30)	0.24 (0.14, 0.34)
2PN oocytes	-0.50 (-0.73, -0.27)	-0.65 (-0.91, -0.38)	0.26 (0.17, 0.35)	0.21 (0.12, 0.31)	0.21 (0.10, 0.33)
2PN cleavage zygotes	-0.48 (-0.71, -0.25)	-0.61 (-0.88, -0.34)	0.26 (0.17, 0.35)	0.22 (0.12, 0.31)	0.22 (0.10, 0.33)
High-quality embryos	-0.71 (-1.03, -0.39)	-0.81 (-1.19, -0.44)	0.27 (0.14, 0.39)	0.29 (0.15, 0.43)	0.32 (0.15, 0.48)
Fertilization rate	-0.07 (-0.49, 0.36)	-0.13 (-0.61, 0.34)	-0.06 (-0.23, 0.11)	-0.07 (-0.26, 0.12)	-0.17 (-0.39, 0.05)
2PN cleavage rate	0.59 (-0.93, 2.11)	1.26 (-0.60, 3.13)	0.00 (-0.60, 0.60)	0.12 (-0.52, 0.77)	0.19 (-0.52, 0.91)
High-quality embryo rate	-0.48 (-0.94, -0.03)	-0.39 (-0.89, 0.11)	0.02 (-0.16, 0.20)	0.16 (-0.04, 0.36)	0.18 (-0.05, 0.41)
**RR (95% CI)**					
Implantation success	0.22 (0.06, 0.87)	0.19 (0.04, 0.80)	1.28 (0.79, 2.08)	1.44 (0.86, 2.40)	1.48 (0.80, 2.73)
Clinical pregnancy	0.24 (0.06, 0.90)	0.15 (0.03, 0.66)	1.39 (0.86, 2.22)	1.49 (0.89, 2.47)	1.71 (0.93, 3.16)
Live birth	0.33 (0.09, 1.17)	0.24 (0.06, 1.00)	1.28 (0.80, 2.05)	1.33 (0.80, 2.21)	1.40 (0.76, 2.58)

[a] All models were adjusted by age (continuous), body mass index (BMI, < 25.0 kg/m^2 vs. ≥25.0 kg/m^2), passive smoking status (yes vs. no), alcohol status (never vs. ever/current), educational level (less than high school vs. high school and above), income (≤5000 vs. >5000 yuan/month) and infertility diagnosis (female factor, male factor, mixed factor vs. unexplained).

Figure S1 Participants' selection flowchart.

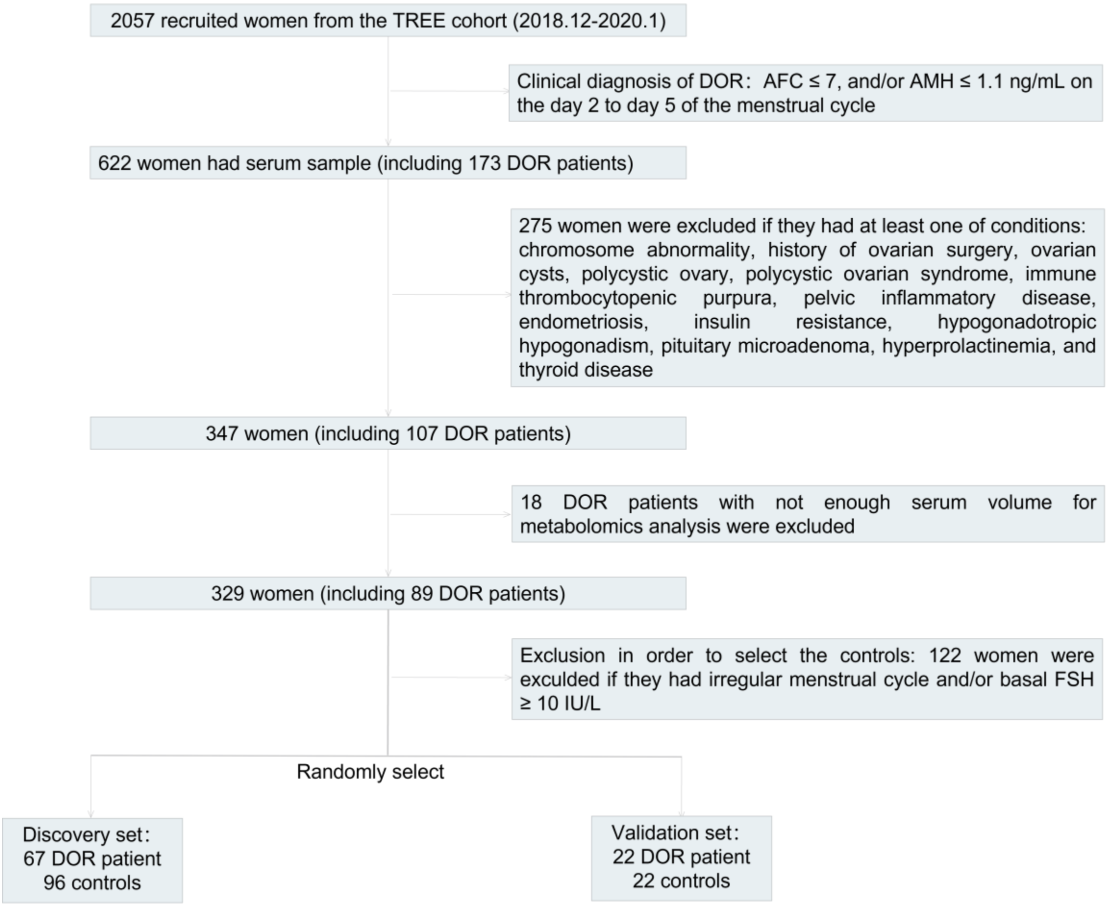

Figure S2 Cross-validation plot with a permutation test repeated 200 times of the OPLS-DA score plot.

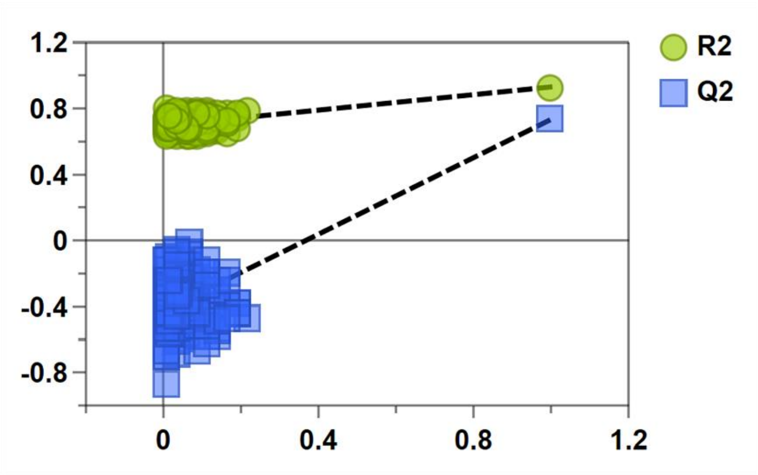

Figure S3 Hierarchical clustering of each sample data set showing the differentially expressed metabolites.

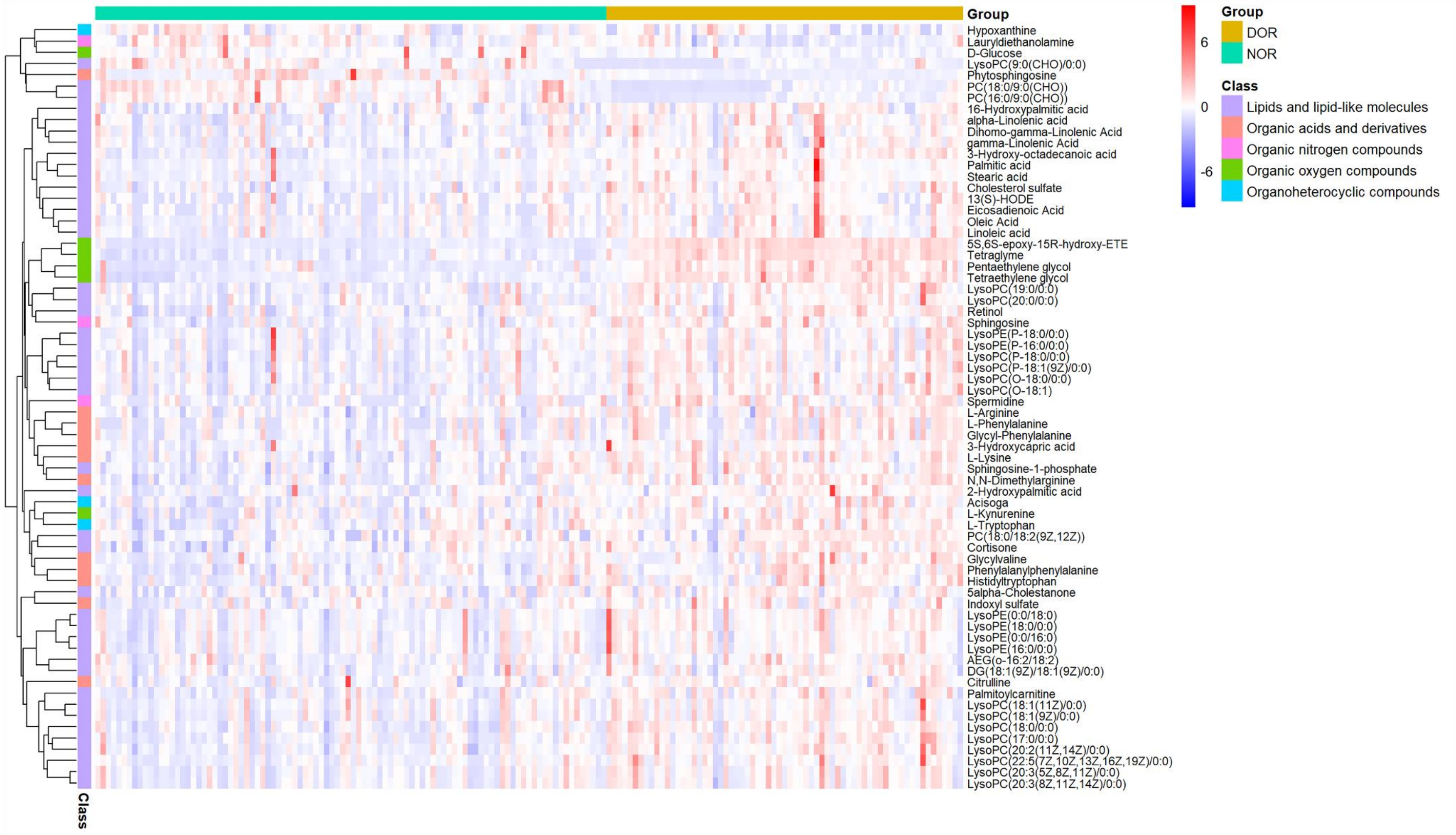

Figure S4 Validation of potential metabolic biomarkers for the diagnosis of DOR. Score plots of PCA (A) and OPLS-DA (B) based on DOR and NOR in the validation set. (C) Cross-validation plot with a permutation test repeated 200 times of the OPLS-DA score plot. The pink circles represent DOR; the blue circles represent NOR; the green circles represent QC. (D) Volcano plot, down-regulated and up-regulated metabolites in DOR compared to NOR are marked in blue and red, respectively.

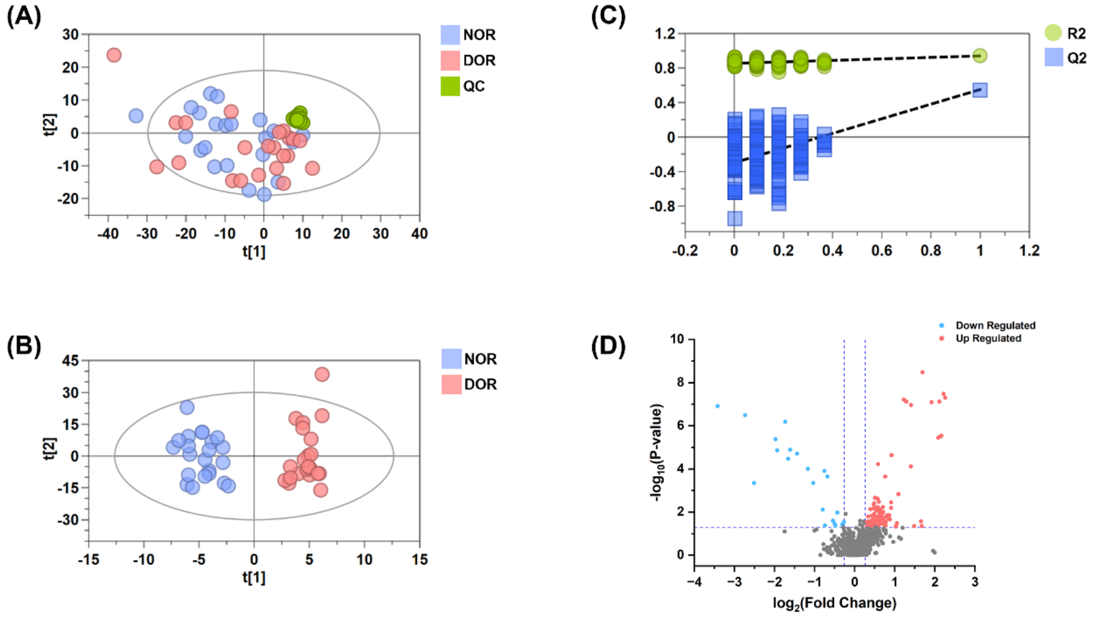

Figure S5 Boxplots of the relative abundances of sphingosine-1-phosphate in the DOR and NOR groups within the discovery set. ***, $P < 0.001$.

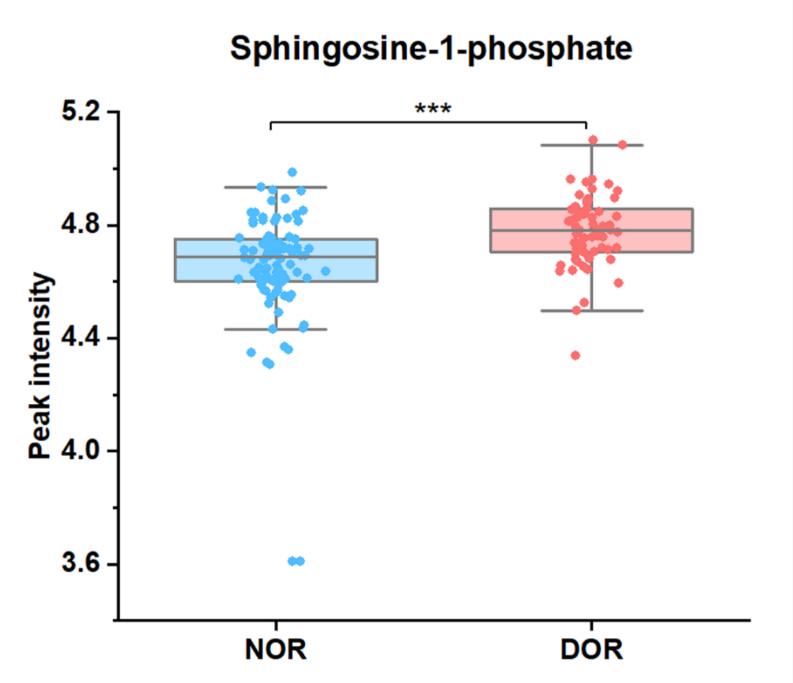

Figure S6 Boxplots of the relative abundances of L-arginine (A1), L-lysine (A2), L-phenylalanine (A3), and L-tryptophan (A4) in the DOR and NOR groups within the discovery set. **, 0.001 < P < 0.01; ***, P < 0.001.

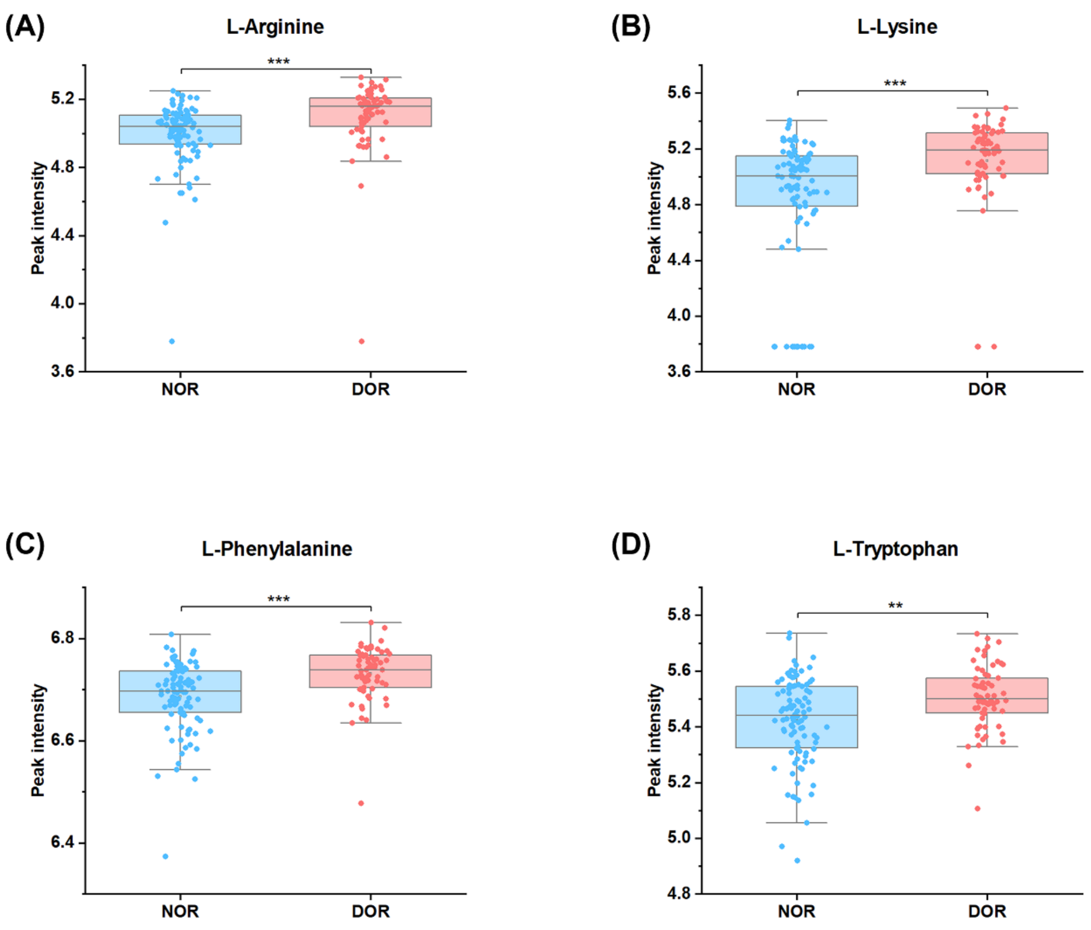

Supplement: Supplementary file 1 [file metabolites-14-00143-s001.zip › metabolites-2869019-supplementary.pdf]
